# How I treat recurrent pediatric high-grade glioma (pHGG): a Europe-wide survey study

**DOI:** 10.1007/s11060-023-04241-6

**Published:** 2023-02-01

**Authors:** Thomas Perwein, Barbara Giese, Gunther Nussbaumer, André O. von Bueren, Miriam van Buiren, Martin Benesch, Christof Maria Kramm

**Affiliations:** 1 Division of Pediatric Hemato-Oncology, Department of Pediatrics and Adolescent Medicine, Medical University of Graz, Auenbruggerplatz 34/2, 8036 Graz, Austria; 2 Department of Pediatrics, Obstetrics and Gynecology, Division of Pediatric Hematology and Oncology, University Hospital of Geneva, Geneva, Switzerland; 3 Cansearch Research Platform for Pediatric Oncology and Hematology, Faculty of Medicine, Department of Pediatrics, Gynecology and Obstetrics, University of Geneva, Geneva, Switzerland; 4 Department of Pediatric Hematology and Oncology, Center for Pediatrics, Medical Center, University of Freiburg, Freiburg, Germany; 5 Division of Pediatric Hematology and Oncology, University Medical Center Göttingen, Göttingen, Germany

**Keywords:** Survey, Treatment, Recurrent, Progressing, Pediatric HGG

## Abstract

**Purpose:**

As there is no standard of care treatment for recurrent/progressing pediatric high-grade gliomas (pHGG), we aimed to gain an overview of different treatment strategies.

**Methods:**

In a web-based questionnaire, members of the SIOPE-BTG and the GPOH were surveyed on therapeutic options in four case scenarios (children/adolescents with recurrent/progressing HGG).

**Results:**

139 clinicians with experience in pediatric neuro-oncology from 22 European countries participated in the survey. Most respondents preferred further oncological treatment in three out of four cases and chose palliative care in one case with marked symptoms. Depending on the case, 8–92% would initiate a re-resection (preferably hemispheric pHGG), combined with molecular diagnostics. Throughout all case scenarios, 55–77% recommended (re-)irradiation, preferably local radiotherapy > 20 Gy. Most respondents would participate in clinical trials and use targeted therapy (79–99%), depending on molecular genetic findings (*BRAF* alterations: BRAF/MEK inhibitor, 64–88%; *EGFR* overexpression: anti-EGFR treatment, 46%; *CDKN2A* deletion: CDK inhibitor, 18%; S*MARCB1* deletion: EZH2 inhibitor, 12%). 31–72% would administer chemotherapy (CCNU, 17%; PCV, 8%; temozolomide, 19%; oral etoposide/trofosfamide, 8%), and 20–69% proposed immunotherapy (checkpoint inhibitors, 30%; tumor vaccines, 16%). Depending on the individual case, respondents would also include bevacizumab (6–18%), HDAC inhibitors (4–15%), tumor-treating fields (1–26%), and intraventricular chemotherapy (4–24%).

**Conclusion:**

In each case, experts would combine conventional multimodal treatment concepts, including re-irradiation, with targeted therapy based on molecular genetic findings. International cooperative trials combining a (chemo-)therapy backbone with targeted therapy approaches for defined subgroups may help to gain valid clinical data and improve treatment in pediatric patients with recurrent/progressing HGG.

**Supplementary Information:**

The online version contains supplementary material available at 10.1007/s11060-023-04241-6.

## Introduction

Pediatric high-grade gliomas (pHGG) are rare, and their biology differs from their adult counterparts [1–3]. This has been considered in the 2021 WHO classification of CNS tumors by introducing the subgroups of pediatric-type diffuse high-grade gliomas and *H3K27*-altered diffuse midline glioma (DMG) [4]. Widely used first-line treatment concepts combine maximal safe resection and local radiotherapy (54–59.4 Gy) with concomitant and adjuvant chemotherapy (mainly temozolomide) for supratentorial pHGG, radiotherapy with or without adjuvant treatment for DMG, and chemotherapy without radiotherapy following resection in very young children [1, 2, 5–11].

In the past decade, new molecular and epigenetic insights, as well as innovative targeted and immunotherapeutic concepts have extended the armamentarium of available treatment options. Yet, the prognosis of pHGG remains dismal, with relapse following initial treatment in the vast majority of patients [1, 2, 6–8, 10, 12–17].

Recurrent/progressing pHGG are virtually incurable, thus usually indicating a palliative treatment situation: Despite multiple efforts and trials investigating new therapeutic approaches, median survival following recurrence is a few months, and there is no established standard of care regimen since many centers pursue a different strategy, most patients undergoing multimodality treatment with very limited success [18–20].

Hence, the present Europe-wide online survey study was set up to collect different views and concepts on treatment options and goals in children and adolescents with recurrent/progressing HGG in representative real-world treatment situations. As the target group of respondents, clinicians with distinct experience in pediatric neuro-oncology were chosen.

## Methods

The conceived case scenarios were intended to represent real-world treatment situations of patients with typical non-DMG diffuse pHGG, DMG, and infant HGG from the HIT-HGG database of the Society of Pediatric Oncology and Hematology in Germany, Austria, and Switzerland (GPOH).

The survey was developed by the authors and tested several times to find a compromise between the broad spectrum of available therapeutic options and an acceptable time effort of approximately 15 min, leaving four representative case scenarios, as portrayed in Table 1.

**Table 1:** Descriptions of the four case scenarios

**Case 1**
A 7-year-old boy underwent total resection of an enhancing parietal lesion. Histopathology disclosed anaplastic astrocytoma WHO grade III. No additional molecular characterization was done. He received 54 Gy/1.8 Gy local radiotherapy with concomitant temozolomide followed by 12 courses of temozolomide maintenance chemotherapy. Three months after the last course, routine follow-up MR imaging showed a progressive enhancing nodule within the radiation field of the primary lesion. At that time, the boy was in good clinical condition without apparent neurological deficits
**Case 2**
An 11-year-old girl was diagnosed with a diffuse midline glioma, *H3.3K27M* (*H3F3A*) mutant, WHO grade IV (DMG), located in the left thalamus, extending into the mesencephalon and obstructing the aqueduct with a resulting hydrocephalus. Endoscopic tumor biopsy and ventriculocisternostomy were performed. After confirmation of DMG by central neuropathological review, radiochemotherapy (59 Gy/1.8 Gy) with temozolomide and valproic acid as HDAC inhibitor and radio- and chemosensitizer was performed. Temozolomide and valproic acid maintenance was continued until the tumor locally progressed 27 months after diagnosis. The tumor was biopsied again via open surgery, and tumor samples were subjected to further molecular work-up. A *BRAFV600E* mutation was found as well as a *SMARCB1* deletion. The girl showed an unaffected clinical condition at relapse with a very good quality of life
**Case 3**
A 17-year-old female was diagnosed with an anaplastic astrocytoma WHO grade III, *IDH1/2* wild-type, after stereotactic biopsy of the tumor lesion. Neuroradiologically, there were typical findings of a gliomatosis cerebri involving the left frontal, parietal, and temporal lobes, both thalami, the right basal ganglia, corpus callosum, mesencephalon and pons. Potentially relevant molecular findings were *EGFR* alterations with marked immunohistochemical *EGFR* expression and a homozygous *CDKN2A* deletion. No MGMT promotor hypermethylation, no *H3K27M*, no *BRAFV600E* or loss of DNA repair enzymes MSH2/MSH6/MLH1/PMS2 were identified. Treatment within HIT-HGG-2013 with temozolomide radiochemotherapy and valproic acid was initiated. Radiotherapy (59 Gy/1.8 Gy) was applied to the whole lesion. Tumor progression was discovered upon maintenance with temozolomide and valproic acid after 8 months. The patient showed marked symptoms with headache, gait disturbance due to a marked dizziness and a loss of weight to persistent nausea, vomiting and loss of appetite
**Case 4**
A 2-year-old boy developed lumbar pain and paraparesis due to a spinal tumor and was subsequently diagnosed by central neuropathological review with an anaplastic glial tumor of high-grade nature, characterized by a high proliferation index of 30% and markedly increased mitotic activity. There was an unresolvable discrepancy to the histological diagnosis by the accompanying molecular work up: A *KIAA1549-BRAF* fusion was found; this fusion and the 850 K methylation array both suggested a pilocytic astrocytoma WHO grade I. Nevertheless, it was decided to treat the patient with three cycles of HIT-SKK chemotherapy, each cycle consisting of four treatment elements with 1 × cyclophosphamide/vincristine, 2 × high dose methotrexate/vincristine, and 1 × carboplatin/etoposide. After chemotherapy, rebiopsy of the residual tumor showed no increased mitotic activity with a proliferation index of 2% indicating no further presence of a high-grade glioma histology. Consequently, no further therapy was applied. Clinically, paraparesis resolved almost completely following first resection. After 8 months of watch-and-wait, the patient showed a radiological relapse with marked spinal dissemination, but without apparent clinical pathology. Re–re-biopsy demonstrated again a high-grade glioma histology with a proliferation index of 5–15%. No molecular work up was performed at that time

The questionnaire was processed in the online survey platform SurveyMonkey® (SurveyMonkey Inc., San Mateo, CA, US). For each case scenario, different options could be chosen from predefined multiple-choice answers. This included assessment of the respondent’s country, profession, and duration of clinical experience, overall treatment goals (curative *versus* palliative care), and finally different treatment options including surgery, radiation, agents, and other modalities, as well as specifications (e.g., extent of resection/radiation dose/agents and combinations). Each question offered an additional free-text option. The questionnaire is provided as supplementary material (Supplementary Material S1).

A short introduction including the link to the survey was sent via e-mail to 212 members of the International Society of Pediatric Oncology Europe-Brain Tumor Group (SIOPE-BTG) and the GPOH. The survey was open for participation from 10 December 2020 until 11 March 2021.

Only responses from participants designated as clinicians experienced in pediatric oncology were considered for detailed data analysis. Data analysis was performed using SurveyMonkey® and GraphPad Prism 9® (GraphPad Software Inc., San Diego, CA, US). Fisher’s exact test was used for comparisons. A p-value of < 0.05 was chosen to indicate statistical significance. Treatment overview graphics were produced using Sankey Diagram Generator v1.2 (Acquire Procurement Services, Brisbane, Australia). Since neither patient data nor human subjects were involved, institutional review board approval was not required for the present study.

## Results

### Participants

143 respondents (SIOPE-BTG, n = 69 [48.3%]; GPOH, n = 74 [51.7%]) from 22 countries participated in the survey. The average duration for completion was 25 min, and the completion rate was 66%. The characteristics of the survey participants are depicted in Table 2. 139 (97%) were identified as clinicians (= clinically active physicians) for detailed analysis. Sixty-nine (49.6%) respondents were from Germany, nine respondents (6.5%) from Italy, seven (5%) were from Switzerland, and six (4.3%) each from France, Spain, and the United Kingdom. Professional experience of more than ten years was indicated by 122 participants (87.8%).

**Table 2:** Epidemiologic characteristics of the 139 survey respondents

	**No. of participants**		**Profession**			**Expertise > 10 years**		**Response rate (%)**
Total	139	100%	PHO	115	82.7%	122	87.8%	66.8
			Pediatrician*	5	3.6%			
			MD*	10	7.2%			
			Neuro-Oncol	1	0.7%			
			Neurosurgeon	1	0.7%			
			Radiotherapist	6	4.3%			
			Biologist	1	0.7%			
*Country of employment*								
Germany	69	49.6%	PHO	52	75.4%	60	87.0%	66.3
			Pediatrician*	5	7.0%			
			MD*	9	13.0%			
*Country of employment*								
Other countries	70	50.4%	PHO	63	90.0%	62	88.6%	67.3
			Neuro-Oncol	1	1.4%			
			MD*	1	1.4%			
Austria	4	2.9%						80.0
Belgium	2	1.4%						66.7
Croatia	1	0.7%						100.0
Czech Republic	1	0.7%						20.0
Denmark	2	1.4%						66.7
France	6	4.3%						54.5
Greece	2	1.4%						100.0
Hungary	2	1.4%						100.0
Iceland	1	0.7%						100.0
Ireland	1	0.7%						100.0
Italy	9	6.5%						90.0
Lithuania	3	2.2%						100.0
Netherlands	5	3.6%						50.0
Norway	2	1.4%						66.7
Portugal	4	2.9%						75.0
Russian Federation	3	2.2%						100.0
Spain	6	4.3%						100.0
Sweden	2	1.4%						50.0
Switzerland	7	5.0%						100.0
Turkey	1	0.7%						100.0
United Kingdom	6	4.3%						29.4

The weblink to the survey was sent via e-mail to 126 members of the International Society of Pediatric Oncology Europe-Brain Tumor Group (SIOPE-BTG) and additionally opened by 104 members of the Society of Pediatric Oncology and Hematology in Germany, Austria, and Switzerland (GPOH)

*Not explicitly declared themselves as pediatric hemato-oncologists in the survey, but members of the SIOPE-BTG or GPOH

*PHO* pediatric hemato-oncologist; *MD* medical doctor

### Responses

Overall, most respondents (90.5–98.5%) would initiate oncological treatment in three out of four case scenarios (Table 3). In Case 3 with gliomatosis cerebri and reduced general condition, palliative/best supportive care was deemed appropriate by 55.8% of experts, while 44.2% still saw an option for oncological treatment.

**Table 3:** Treatment proposals by the survey respondents (n = 139)

**Treatment**	**Answers total**	**Case 1**	**Case 2** ^d^	**Case 3** ^e^	**Case 4** ^f^
Respondents	n = 139	n = 133	n = 104	n = 95	n = 95
Palliative care	16.4%	1.5%	5.8%	**55.8%**	9.5%
Oncological treatment	**83.6%**	**98.5%**	44.2%	**94.2**%	**90.5%**
Curative intent	44.8%	**62.6%**	16.3%	9.5%	**67.4%**
Phase I/II trials	**88.7%**	**91.7%**	**96.9%**	**76.2%**	**81.4%**
*Surgery*	43.4%	**92.4%**	8.3%	23.8%	24.4%
Maximal safe resection	**79.9%**	**90.0%**	**75.0%**	40.0%	47.6%
Biopsy	20.1%	10.0%	25.0%	**60.0%**	**52.4%**
Molecular workup	**95.3%**	**96.4%**	**100%**	**100%**	**85.7%**
*(Re-) Irradiation*	**69.5%**	**76.9%**	**72.9%**	**54.8%**	**62.8%**
Local radiotherapy	**81.3%**	**97.7%**	**94.3%**	**87.0%**	35.2%
Craniospinal radiotherapy	18.7%	2.3%	5.7%	13.0%	**64.8%**
Dose > 20 Gy	**67.7%**	**65.9%**	**61.4%**	**60.9%**	**81.5%**
*Chemotherapy*	**57.6%**	**71.7%**	30.9%	**54.8%**	**69.8%**
Lomustine (CCNU)	17.1%	22.2%	13.8%	34.8%	5.0%
PCV	7.8%	11.1%	3.4%	17.4%	1.7%
Temozolomide	19.2%	11.1%	10.3%	8.7%	38.3%
Etoposide + Trofo. oral	7.8%	8.6%	10.3%	8.7%	5.0%
Other Chemotherapy^a^	42.0%	37.0%	44.8%	21.7%	**55.0%**
CT plus HDAC inhibitors	12.4%	14.8%	13.8%	4.3%	11.7%
*Targeted therapy*	**89.4%**	**93.8%**	**98.9%**	**78.6%**	**78.8%**
Depending on results/trials	39.0%	**81.0%**	6.7%	15.2%	28.4%
BRAF/MEK inhibitor	44.4%	8.6%	**87.8%**	-	**64.2%**
Anti EGFR (TKI/nimotuzumab)	7.5%	4.8%	2.2%	45.5%	-
EZH2 inhibitor	3.7%	-	12.2%	-	-
CDK inhibitor	2.0%	-	-	18.2%	-
Other targeted therapy^b^	9.5%	13.3%	6.7%	9.1%	7.5%
Bevacizumab	12.2%	16.2%	5.6%	18.2%	11.9%
*Immunotherapy*	43.3%	**69.1%**	39.6%	31.0%	20.0%
Checkpoint inhibitor	30.3%	38.2%	27.8%	15.4%	11.8%
Tumor vaccine	16.2%	21.1%	11.1%	15.4%	5.9%
Other immunotherapy^c^	4.9%	6.5%	2.8%	7.7%	-
*Tumor-Treating Fields*	15.9%	26.4%	15.6%	19.0%	1.2%
*Intraventricular CT*	12.9%	13.8%	4.4%	7.1%	23.5%

Chosen answers and free-text proposals by the 139 respondents. Percentage is related to the respective number of answers for each question. Bold font indicates majority

^a^Oral topotecan/etoposide ± cyclophosphamide, 7.3%; modified HIT-SKK, 5.2%; irinotecan + bevacizumab, 4.1%; vinblastine, 3.6%; oral antiangiogenetic combination, 3.6%; “metronomic chemotherapy” not specified, 2.1%; HIT-HGG-Rez-Immunovac study, 2.1%; LGG chemotherapy (carboplatin, vincristine), 2.1%; vincristine or vinorelbine, 2.1%; PEI (cisplatin, etoposide, ifosfamide), 1.6%; carboplatin + etoposide, 1.6%; High-dose chemotherapy + autologous stem cell transfusion, 1.0%; RIST (rapamycin, irinotecan, dasatinib, temozolomide), 1.0%; ICE (ifosfamide, carboplatin, etoposide), 1.0%; carboplatin-based, 0.5%; eribulin, 0.5%; fotemustine, 0.5%; Metro-PD1, 0.5%; adriamycine, 0.5%; vindesine, 0.5%, VAC (vincristine, actinomycin-d, cyclophosphamide), 0.5%;

^b^TRK inhibitor, 2.0%; DRD2 inhibitor (ONC 201), 2.0%; mTOR inhibitor, 2.0%; multi TKI, 1.4%; Hedgehog inhibitor, 0.7%; PDGFR inhibitor, 0.3%; PIK3CA inhibitor, 0.3%; PARP inhibitor, 0.3%; ALK inhibitor, 0.3%;

^c^CAR-T cells, 3.5%; Oncolytic virus, 1.4%;

^d^*BRAFV600E* mutation and *SMARCB1* deletion;

^e^*EGFR* overexpression and *CDKN2A* deletion;

^f^*KIAA1549-BRAF* fusion

*PCV* procarbazine + CCNU + vincristine; *Trofo* trofosfamide; *CT* chemotherapy; *TKI* tyrosine kinase inhibitor

In Case 1, 74.4% of participants would suggest invasive diagnostic and/or therapeutic measures, as would 50% in Case 4. In Case 2 and Case 3, 75% of respondents, and 85.7% of experts would only accept a favorable toxicity and/or stress profile, as well as predominant outpatient treatment (75% and 69.1%), respectively.

**Fig. 1:**
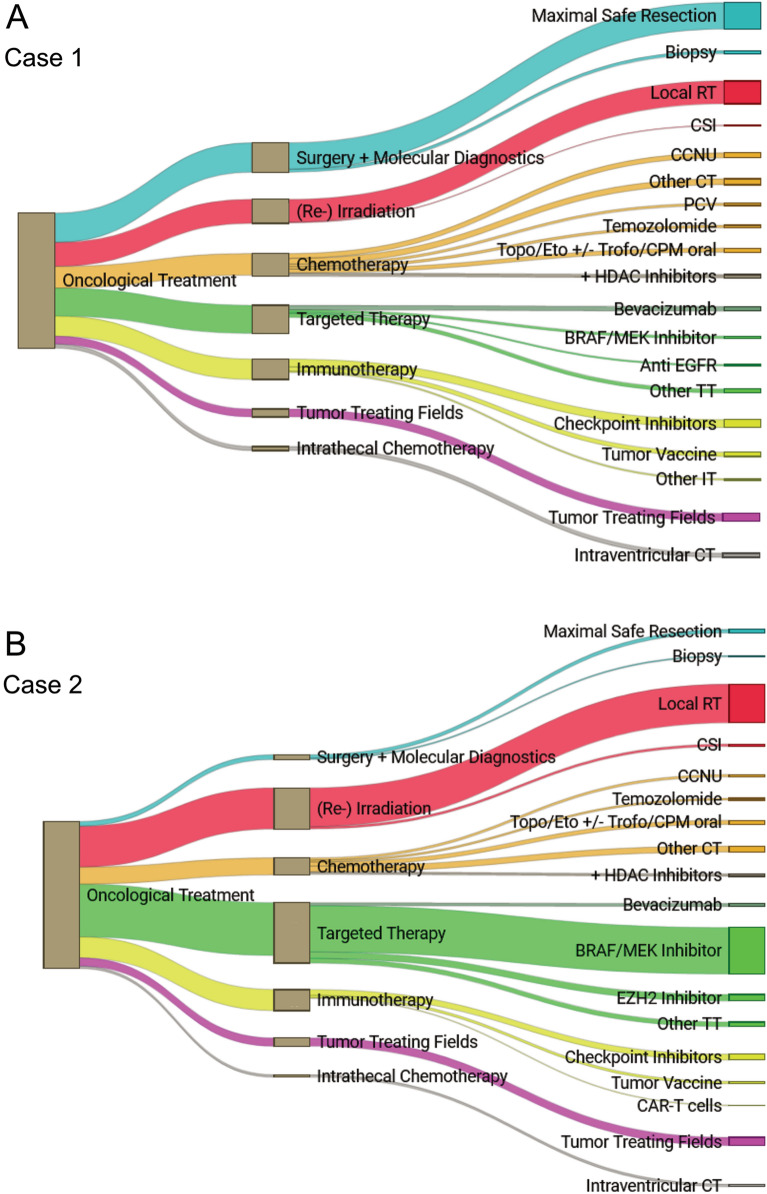

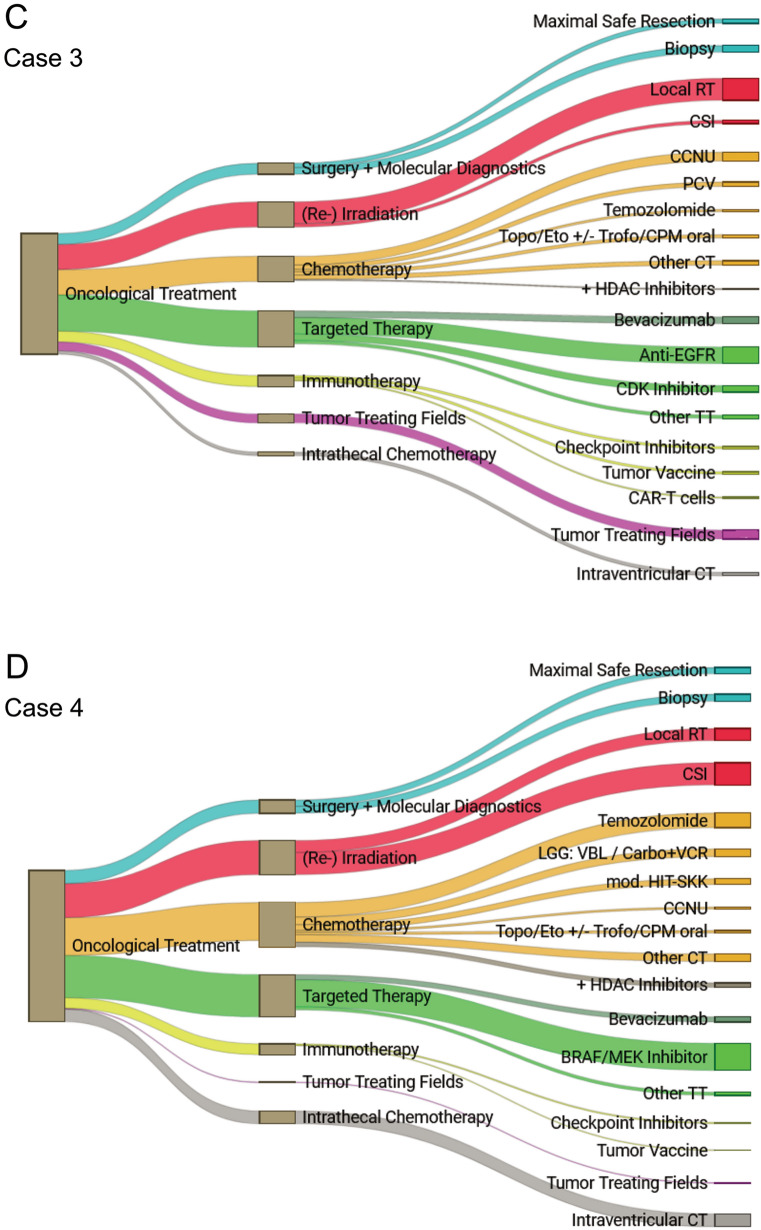
Oncological treatment options proposed by survey respondents. Oncological treatment options chosen and proposed by the 139 survey respondents for Case 1 (**A**), Case 2 (**B**), Case 3 (**C**) and Case 4 (**D**). *Abbreviations: RT* radiotherapy; *CSI* craniospinal irradiation; *CCNU* lomustine; *CT* chemotherapy; *PCV* procarbazine, *CCNU* vincristine; *Topo* topotecan; *Eto* etoposide; *Trofo* trofosfamide; *CPM* cyclophosphamide; *TT* targeted therapy; *IT* immunotherapy; *VBL* vinblastine; *Carbo* + *VCR* carboplatin, vincristine

Throughout all case scenarios, 76.2–96.9% of the respondents considered an option to participate in phase I/II clinical trials. An overview of the proposed treatment for each case scenario (Cases 1–4) is provided in Table 3 and Fig. 1.

Re-resection was regarded as a therapeutic option by 92.4% in Case 1 with parietal anaplastic astrocytoma, the majority (90%) intending maximal safe resection.

Most participants (75.6%–91.7%) objected to re-surgery in the other three cases (Cases 2, 3, 4). Experts favoring surgery mainly chose biopsy (52.4–60%) in Cases 3 and 4. Throughout all cases, 85.7–100% of experts would initiate a molecular genetic profiling of the obtained tumor tissue.

In all four cases, most respondents (54.8–76.9%) would include (re-)irradiation in their therapeutic strategy, preferably (60.9–81.5%) exceeding 20 Gy up to the maximal applicable dose according to the appraisal of a radio-oncologist (Table 3). In cases 1, 2 and 3, local re-irradiation was contrived (87–97.7%). In Case 4 (young child with disseminated spinal pHGG), 64.8% of experts favoring radiotherapy suggested craniospinal irradiation (Table 3, Fig. 1).

Chemotherapy was considered in 30.9–71.7%, and chosen by a majority in all case scenarios, except for Case 2 with DMG (Table 3). Overall, the most common agents proposed were CCNU (17.1%) as monotherapy or in combination with bevacizumab or temozolomide, or additionally as part of the combinatory regimen PCV (procarbazine/CCNU/vincristine; 7.8%), followed by temozolomide (19.2%) as monotherapy or combination with CCNU or valproic acid. In general, 12.4% of respondents would add histone deacetylase (HDAC) inhibitors (e.g., valproic acid, panobinostat, vorinostat, or entinostat).

In Case 1, CCNU (22.2%) and HDAC inhibitors (14.8%) were preferred, followed by PCV and temozolomide (each 11.1%). Of the trial participants approving chemotherapy in Case 2 with DMG (30.9%), 13.8% each proposed CCNU, oral topotecan or etoposide with/without cyclophosphamide, and HDAC inhibitors. Chemotherapy with CCNU (34.8%) or PCV (17.4%), was most frequently suggested in Case 3. Temozolomide (38.3%), or additional modified HIT-SKK cycles (15%) were the preferred regimens in Case 4, and 11.7% would add HDAC inhibitors.

In each case, targeted therapy was proposed by a majority of 78.6–98.9% experts, depending on molecular findings and/or availability of clinical trials. The most popular targeted agents (44.4%) were BRAF inhibitors (e.g., dabrafenib) and MEK inhibitors (e.g., trametinib), preferably in combination, followed by *EGFR*-directed treatment (7.5%), either with tyrosine kinase receptor inhibitors (TKIs, e.g., erlotinib) or with monoclonal antibodies (nimotuzumab). 12% supported the use of bevacizumab.

In absence of a molecular genetic profile for the first case scenario, 81% of respondents stated that they would choose targeted treatment depending on molecular findings and/or availability of clinical trials. Bevacizumab was proposed by 16.2% in Case 1.

In Case 2, with the described alterations of *H3K27M, BRAFV600E* and *SMARCB1*, BRAF/MEK inhibitor treatment, preferably as combined treatment (e.g., dasatinib/trametinib), was favored (87.8%). A total of 12.2% of respondents would administer EZH2 inhibitors (tazemetostat).

In Case 3 with reported *EGFR* overexpression and *CDKN2A* deletion, anti-*EGFR* treatment was suggested by 45.5% of experts, using TKI (e.g., erlotinib) or nimotuzumab. 18.2% of respondents conceived CDK inhibitors (e.g., palbociclib), and another 18.2% thought of using bevacizumab.

In Case 4, participants preferably recommended targeting the described *KIAA1549-BRAF* fusion with BRAF/MEK inhibitors (64.2%), mainly with MEK inhibitors such as trametinib. 28.4% of respondents would base their decision upon actual molecular genetic findings. Bevacizumab was proposed by 11.9% of experts.

In Case 1, immunotherapeutic options were considered in the context of available early phase trials by most experts (69.1%), also dependent on their availability in the respective country. In the other three case scenarios, immunotherapy was declined by most participants (60.4–80%). Throughout all case scenarios, most treating physicians conceiving immunotherapy would apply checkpoint inhibitors (30.3%) such as nivolumab, either alone, or in combination with entinostat or tumor vaccines. Tumor vaccines, mainly based on dendritic cell vaccines, were the second most common option for immunotherapy (16.2%).

Overall, 15.6–26.4% of respondents would apply tumor-treating fields (TTF) in the three cases with intracranial HGG (Table 3, Fig. 1).

In Case 4 with disseminated intraspinal HGG, 23.5% of experts would administer intraventricular chemotherapy (etoposide, topotecan, methotrexate and/or cytarabine) as part of a multimodal treatment concept. This applied for 4.4–13.8% in the other three case scenarios (Table 3, Fig. 1).

Altogether, the preferred combination of treatment modalities chosen in Case 1 with a hemispheric diffuse HGG was maximal safe resection, reirradiation, targeted treatment, chemotherapy, and immunotherapy (with or without tumor-treating fields).

In Case 2 with *H3K27M-*mutated DMG (and *BRAFV600E* and *SMARCB1* alterations), and Case 3 with gliomatosis cerebri (*EGFR* overexpression and *CDKN2A* deletion) in reduced clinical condition, local re-irradiation and targeted therapy (with or without chemotherapy and/or immunotherapy) were proposed.

The preferred treatment combination chosen in Case 4, a young child with disseminated spinal cord HGG (*KIAA1549-BRAF* fusion), was targeted treatment, chemotherapy, and craniospinal irradiation (with or without intraventricular chemotherapy).

As presented in Supplementary Table S3, several chosen strategies differed significantly between German participants and experts from other European countries: Respondents from Germany preferred oral etoposide/trofosfamide (15.6% vs. 1%, p = 0.0002), modified HIT-SKK chemotherapy (8.9% vs. 1.9%, p = 0.0472) and HDAC inhibitors (21.1% vs. 3.9%, p = 0.0002), as well as the addition of chloroquine (5.6% vs. 0%, p = 0.0208), while barely choosing PCV chemotherapy (1.1% vs. 13.6%, p = 0.0009). They less often considered bevacizumab (3.0% vs. 19.8%, p < 0.0001) or irinotecan plus bevacizumab (0% vs. 7.8%, p = 0.0076), and more often favored checkpoint inhibitors (39.7% vs. 17.4%, p = 0.0051) and/or tumor vaccines (26% vs. 5.8%, p = 0.0012), as well as intraventricular chemotherapy (17.5% vs. 9%, p = 0.0305).

## Discussion

With the present survey, we aimed to obtain an overview of different therapeutic strategies in children and adolescents with recurrent/progressing pHGG across different European countries by interviewing experts in pediatric neuro-oncology on their advice in four case scenarios chosen to represent routine treatment situations in pHGG.

Across the 22 European countries and all case scenarios, most respondents considered it important to include pHGG patients in phase I/II clinical trials at recurrence or progression. Currently, there are 56 ongoing early phase trials (mainly at US sites) including pediatric patients with recurrent/progressing HGG registered at ClinicalTrials.gov or the EU Clinical Trials Register, most of them focusing on targeted treatment or immunotherapeutic approaches (Supplementary Table S2). Although such trials are not equally available in all countries, probably due to regulatory issues, this reflects the vigorous worldwide efforts towards finding urgently needed innovative and effective treatment approaches in this challenging setting. It also exhibits the scientific and clinical willingness for cooperation, studying concerted treatment concepts rather than pursuing mere individual decisions and strategies.

Most survey participants intended oncological treatment in three out of four case scenarios, while in the case of a 17-year-old female with gliomatosis cerebri and marked symptoms, more than half of respondents chose palliative treatment/best supportive care, highlighting the importance of considering health condition in the therapy concept. The widespread intention observed in the present survey to continue oncological treatment of young patients facing the (except in case four) most likely palliative situation of recurrent/refractory HGG may also reflect the hope and strive of treating physicians to find an effective treatment in the near future. However, the real-life status quo of patients and their families often contrasts these efforts: Their needs and wishes are the mainstay of best supportive care, which should be addressed and planned early enough at disease recurrence/progression.

Maximal safe resection was consensually considered in only one case of localized hemispheric HGG. While extended resection is impossible in gliomas of the brainstem, basal ganglia and thalamus, a higher extent of resection is known to be a positive prognostic factor for survival in non-pontine pHGG at initial diagnosis and in adult patients with recurrent glioblastoma [1, 6, 12, 21]. In DMG, biopsy has been shown to be feasible, has contributed especially to recent knowledge on the biology, and has therefore been recommended to gain information on potentially druggable targets whenever practicable [8, 23, 24].

Throughout all case scenarios, radiotherapy was considered as a backbone of a multimodal relapse treatment, with most experts proposing local re-irradiation up to the maximal applicable dose in patients with localized supratentorial tumors, DMG, or gliomatosis cerebri. In a meta-analysis of therapeutic approaches spanning the past 20 years in pediatric patients with recurrent non-pontine HGG, Kline et al. found an overall survival of 14 months with local re-irradiation, which was longer than with other approaches [18]. Re-irradiation with 30–54 Gy led to clinical and survival improvement in pHGG patients including DIPG, especially when the interval between the first and second irradiation was longer than one year [8, 19, 20, 24, 25]. Although the role of craniospinal irradiation in disseminated disease remains unclear and hematological toxicity has to be expected, its feasibility has been reported [26]. In the present survey, two thirds of experts recommended CSI (preferably after deferral through chemotherapy) in a young child with disseminated spinal cord infant HGG.

Systemic chemotherapy was chosen by up to three quarters of experts in non-DMG cases in the present survey, while it was regarded as promising by barely one third in DMG. Temozolomide was considered the first option for relapse treatment in one temozolomide-naïve case (Case 4). CCNU (either as monotherapy or in combination) was the preferred agent in patients who had already received initial radiochemotherapy with temozolomide. The addition of CCNU to temozolomide maintenance treatment improved survival in pHGG and CCNU is commonly used in adult patients with recurrent glioblastoma, limited however, by considerable hematological toxicity [6, 27].

Numerous combination chemotherapy strategies have been tested in patients with pHGG, lacking obvious survival advantages with any particular regimen, but yielding increased toxicity with intensified combinations [8, 9, 13, 14, 16, 28–32]. In the meta-analysis conducted by Kline et al., chemotherapy approaches showed an overall survival of only four months in pediatric patients with recurrent HGG [18]. The international differences observed in the present study concerning some proposed regimens additionally highlight this lack of clearly superior chemotherapy approaches in recurrent pediatric HGG.

In selected young children with HGG, a first-line chemotherapy approach allows deferring or even avoiding radiotherapy and may therefore be preferred in “infant HGG” [11, 33]. The clinical impact of targeted therapy in case of, e.g., *ALK, ROS* or *NTRK* gene fusions is still subject of ongoing clinical studies. Despite reports of impressive clinical responses, the duration of tumor control and the best possible application mode and time point of these inhibitors in addition to or as substitution for chemotherapy remain unclear.

Intraventricular chemotherapy was recommended as part of a systemic chemotherapy regimen or metronomic antiangiogenetic treatment concept by a quarter of respondents in Case 4 with disseminated spinal cord HGG.

More than ten percent of experts would add epigenetic modifier treatment with histone deacetylase (HDAC) inhibitors, e.g., valproic acid, which may sensitize HGG to radiochemotherapy. Retrospective analysis suggested better outcomes with valproic acid as antiepileptic treatment in pHGG patients including DIPG [17, 34]. A recent phase II study of valproate added to radiotherapy and bevacizumab in newly diagnosed pHGG observed tumor responses and indicated a survival benefit for patients with glioblastoma and constitutive mismatch repair deficiency, although in total, EFS and OS were not improved compared with historical data [10]. In the ongoing HIT-HGG-2013 trial, valproate is added to radiotherapy and temozolomide in children and adolescents with HGG (EudraCT: 2013-004,187-56).

Almost all European experts agreed on the importance of a molecular genetic workup to possibly identify alterations allowing for targeted treatment, which was regarded as mainstay of treatment in all cases in the present survey.

BRAF/MEK inhibition was the most popular targeted treatment option among survey participants. It has been reported to induce responses in *BRAFV600E*-mutated pHGG and is likely to be more effective in a combination regimen [1, 33, 35–37]. The combination of dabrafenib and trametinib in children with recurrent/progressing HGG is currently being investigated in a phase II trial (NCT02684058; Supplementary Table S2).

Other targeted treatment options proposed in the present survey were EGFR-directed treatment (monoclonal antibody nimotuzumab or TKI erlotinib) in case of *EGFR* overexpression, CDK inhibitors in case of *CDKN2A* deletion and EZH inhibitors for *H3K27M* mutated DMG with *SMARCB1* deletion.

Druggable targets can be found in a considerable proportion of patients with pHGG, and personalized treatment concepts may offer benefits for selected patients, where prolonged survival and tumor responses can be observed, even in DIPG [8, 14, 15, 22, 24, 33, 37–39]. However, apart from high-priority targets such as *BRAF-, ALK-, ROS-* or *NTRK-*alterations, evidence is still limited for most therapeutic options. Although there is no clear survival advantage so far and the spectrum of potential targeting drugs crossing the blood–brain-barrier is narrow to date, most experts exhibited their expectations on targeted treatment in the present study.

The addition of the VEGF-directed monoclonal antibody bevacizumab to (radio-) chemotherapy showed some objective responses in a subset of pediatric HGG patients (e.g., with contrast-enhancing lesions more similar to “adult” patterns) but failed to improve survival, at the same time increasing toxicity [10, 40–42]. Nevertheless, adult data show that the addition of bevacizumab in recurrent glioblastoma can yield a survival benefit, may spare steroids and stabilize the clinical status for a longer time due to its anti-edematous effect, and, despite its toxicity, has no negative effect on quality of life [43]. This may be reflected in the recommendations by European pediatric neurooncologists in the present survey study, where bevacizumab was chosen by twelve percent of respondents.

Immunotherapeutic approaches were considered by a significant proportion of respondents only in Case 1 of the present survey, and their selection would depend on the availability of clinical trials. This was also reflected by a significant difference in proposing immunotherapy concepts between experts from other European countries and those from Germany, where trials combining metronomic cyclophosphamide and dendritic cell vaccination with checkpoint blockade (nivolumab/ipilimumab) for relapsed HGG (HIT-Rez-Immunovac, NCT03879512, Supplementary Table S2) or checkpoint inhibition (nivolumab) with entinostat in relapsed malignancies (INFORM2-NivEnt, NCT03838042) are currently recruiting.

While, generally, pediatric tumors show a low tumor mutational burden (TMB), limiting efficacy of checkpoint inhibitors, sustained responses were observed in some pHGG patients with germline mismatch repair deficiency or high TMB, especially when using a combined approach [2, 15, 37, 44]. Interim results of the ongoing HIT-Rez-Immunovac trial were promising, but current vaccination strategies require (near) total resection and still lack a clear survival benefit compared to re-irradiation in adults with relapsed HGG [45, 46]. Anti-GD2-directed CAR-T cell application for DMG may be another future option, and local delivery of HER2-directed CAR-T cells is currently being tested in children with refractory/relapsed CNS tumors (BrainChild-01, NCT0350099) [15, 47]. Yet, novel immunotherapeutic approaches still face challenges with crossing the blood–brain-barrier, low mutational burden, tumor heterogeneity, immunological “cold” tumors, and antigen escape [15].

Tumor-treating fields (TTF), chosen by a quarter of respondents in Case 1, yielded improved survival when combined with temozolomide maintenance in adult glioblastoma patients [48]. Despite toils like its long daily application time, TTF was reported to be feasible and well-tolerated even in young pediatric patients [49, 50], and is subject of ongoing trials (see Supplementary Table S2).

Our survey implied some limitations. First, the predefined answers may have simplified the clinical decision-making. To allow more individualized and detailed statements, additional free-text options were provided. However, the analysis of the free-text answers may be hampered and potentially biased by our attempts to categorize them for comparison and interpretation. Moreover, the respondents had to rely only on the information provided in the case descriptions, without the opportunity for additional information and/or discussion with colleagues. Decision-making might have been easier if we had provided imaging or detailed radio-morphologic descriptions to obtain an additional visual impression or rule out signs of pseudo-progression, for example.

The overrepresentation of one country (Germany) may be another limitation of the study. Beside Germany’s large population, this is because the weblink was provided not only to members of the SIOPE-BTG, but also to members of the GPOH in Germany, Austria and Switzerland in order to increase the scope of the survey. Moreover, the response rate from Germany and German-speaking countries was higher than that from other large European countries (e.g., France or UK), which may be partly explained by the circumstance that the survey’s initiators come from within the HIT (Brain Tumor) network of the GPOH.

On the other hand, the fact that half of all respondents came from Germany allowed us to reveal some international differences in treatment practice, which may be due to varying experience with pursued treatment strategies between the respective centers, as well as different accessibility of phase I/II trials. Thus, treatment decisions may have been influenced by the availability of certain treatment modalities in the respective countries and some might also have been based on personal opinions and preferences as well as previous experiences. However, it was not the goal of this study to evaluate medical evidence for the therapeutic strategies chosen by the respondents.

Nevertheless, the high number of respondents highlights an overall need for sharing expertise in the desperate setting of treating young patients with relapsed/refractory HGG and discussing treatment decisions. The fact that all respondents came from European countries being embedded in the SIOPE-BTG network, working in similar professional settings, most of them with a professional experience of more than ten years, enabled comparable and meaningful results to at least identify general tendencies for decision making in such difficult, highly individualized treatment situations.

## Conclusions

To conclude, this international web-based survey presents treatment proposals by more than one hundred experienced professionals for typical scenarios in pediatric patients with recurrent/progressing HGG. It might therefore serve as support for clinicians treating young patients in this challenging situation. Moreover, answers reflect the willingness of pediatric oncologists across Europe to share their expertise to develop joint and multimodal treatment concepts. Thus, our survey might serve as a push towards concerting treatment in the context of international multicenter collaborations, including maximal safe resection (whenever feasible) or biopsy (in DMG, gliomatosis or disseminated tumors) with molecular testing, local re-irradiation up to the maximum tolerable dose (or CSI in disseminated tumors), and adjuvant chemotherapy (preferably with CCNU in patients pre-treated with temozolomide), serving as a backbone for a personalized targeted treatment according to molecular genetic findings and novel immunotherapeutic approaches where prerequisites are fulfilled.

## Supplementary Information

Below is the link to the electronic supplementary material.

Supplementary file1 (PDF 139 KB)

Supplementary file2 (PDF 214 KB)

Supplementary file3 (PDF 177 KB)

## Data Availability

The original data that support the findings of this study are available from the corresponding author, TP, upon reasonable request.
