# Supplementary material for: How I treat recurrent pediatric high-grade glioma (pHGG): a Europe-wide survey study
Source: J Neurooncol. 2023 Feb 1;161(3):525–38. doi: 10.1007/s11060-023-04241-6 (PMC9992031; doi:10.1007/s11060-023-04241-6)
Supplement: Supplementary file 1 — Supplementary file1 (PDF 139 KB) [file 11060_2023_4241_MOESM1_ESM.pdf]

Supplementary Material S1. Questionnaire template for the online survey.

- **Please select your country of employment:**
- **Profession:**
- **Duration of professional experience:**

☐ < 10 years
☐ > 10 years

- **How would you treat this patient?**

1) Please define YOUR therapeutic goals:

a. <u>Oncological treatment</u>

☐ With curative intent

☐ With non-curative intent

b. <u>No further oncological treatment (palliative care/best supportive care)</u>

☐ Yes ☐ No

---

2) Please choose between the following options (single/multiple answers possible)

- Invasive diagnostic and/or therapeutic measures?

☐ Yes ☐ No

- Predominant inpatient therapy acceptable

☐ Yes ☐ No

- Only predominant outpatient therapy acceptable

☐ Yes ☐ No

- Only predominant therapy at home acceptable

☐ Yes ☐ No

- Treatment profile with marked toxicity and/or stress acceptable

☐ Yes ☐ No

- Only favorable toxicity and stress profile acceptable

☐ Yes ☐ No

---

3) Do you see an indication for participation in early clinical trials (phase I/II)?

☐ Yes ☐ No

---

4) Surgery

☐ Yes ☐ No

If yes:

☐ a) Rebiopsy only

☐ b) Maximal safe resection

☐ + Molecular work up

---

5) (Re-)irradiation

☐ Yes ☐ No

If yes:

☐ Only local ☐ Craniospinal

Energy dose:

☐ ≤ 20 Gy

☐ > 20

Please specify:

---

6) Chemotherapy

☐ Yes ☐ No

If yes, please specify:

---

7) Targeted therapy

☐ Yes ☐ No

If yes, please specify:

---

8) Immunotherapy

☐ Yes ☐ No

If yes, please specify:

---

9) Tumor treating fields (e.g. Optune®)

☐ Yes ☐ No

---

10) Intraventricular chemotherapy

☐ Yes ☐ No

If yes, please specify:

---

11) Other treatment options:

☐ Yes ☐ No

If yes, please specify:
