# Supplementary material for: How I treat recurrent pediatric high-grade glioma (pHGG): a Europe-wide survey study
Source: J Neurooncol. 2023 Feb 1;161(3):525–38. doi: 10.1007/s11060-023-04241-6 (PMC9992031; doi:10.1007/s11060-023-04241-6)
Supplement: Supplementary file 2 — Supplementary file2 (PDF 214 KB) [file 11060_2023_4241_MOESM2_ESM.pdf]

Supplementary Table S2: **Ongoing early phase clinical trials including paediatric patients with recurrent/progressing pHGG (n=56)**
(ClinicalTrials.gov, EudraCT; status: ongoing; 8 March 2022)

ID	Phase	Status	Intervention	Type	Country	Link
NCT05096481	Phase 2	Not yet recruiting	PEP-CMV / Tetanus Diphtheria Vaccine / Temozolomide	IT + CT	US	https://ClinicalTrials.gov/show/NCT05096481
NCT03033992		Recruiting	Optune System (NovoTTF-200A System)	TTF	US	https://ClinicalTrials.gov/show/NCT03033992
NCT05082493	Phase 1	Not yet recruiting	Berubicin Hydrochloride	CT		https://ClinicalTrials.gov/show/NCT05082493
NCT04323046	Phase 1	Recruiting	Ipilimumab / Nivolumab / Placebo	IT	US, AU, IL, Europe (CH)	https://ClinicalTrials.gov/show/NCT04323046
NCT03299309	Phase 1	Recruiting	PEP-CMV	IT	US	https://ClinicalTrials.gov/show/NCT03299309
NCT04295759	Phase 1	Recruiting	INCB7839	TT	US	https://ClinicalTrials.gov/show/NCT04295759
NCT03128047	Phase 1	Recruiting	Optune NovoTTF-200A System	TTF	US	https://ClinicalTrials.gov/show/NCT03128047
NCT02359565	Phase 1	Recruiting	Pembrolizumab	IT	US	https://ClinicalTrials.gov/show/NCT02359565
NCT04482933	Phase 2	Not yet recruiting	Biological G207	IT	US	https://ClinicalTrials.gov/show/NCT04482933
NCT04978727	Phase 1	Active, not recruiting	SurVaxM	IT	US	https://ClinicalTrials.gov/show/NCT04978727
NCT04201457	Phase 1/2	Recruiting	Dabrafenib / Trametinib / Hydroxychloroquine	TT + OTHER	US	https://ClinicalTrials.gov/show/NCT04201457
NCT03690869	Phase 1/2	Recruiting	Cemiplimab (monotherapy) / Irradiation	IT + RT	US	https://ClinicalTrials.gov/show/NCT03690869
NCT04254419	Phase 1	Not yet recruiting	NK cells	IT	US	https://ClinicalTrials.gov/show/NCT04254419
NCT03879512	Phase 1/2	Recruiting	Depletion of regulatory T cells / Surgery / Cancer vaccine / Checkpoint blockade	IT + OP	Europe (DE)	https://ClinicalTrials.gov/show/NCT03879512
NCT03043391	Phase 1	Active, not recruiting	Polio/Rhinovirus Recombinant (PVSRIPO)	IT	US	https://ClinicalTrials.gov/show/NCT03043391
NCT02684058	Phase 2	Active, not recruiting	Dabrafenib / Trametinib / Carboplatin / Vincristine	TT + CT	US, CA, AR, BR, AU, IL, JP, Europe (BE, CZ, DK, FI, FR, DE, IT, NL, RU, ES, SE, CH, GB)	https://ClinicalTrials.gov/show/NCT02684058
NCT02323880	Phase 1	Active, not recruiting	Selinexor	TT	US	https://ClinicalTrials.gov/show/NCT02323880
NCT03598244	Phase 1	Recruiting	Savolitinib	TT	US	https://ClinicalTrials.gov/show/NCT03598244
NCT02885324	Phase 2	Recruiting	Cabozantinib	TT	US	https://ClinicalTrials.gov/show/NCT02885324
NCT01130077	Phase 1	Active, not recruiting	HLA-A2 restricted glioma antigen peptides vaccine / Poly-ICLC	IT	US	https://ClinicalTrials.gov/show/NCT01130077
NCT03893487	Early Phase 1	Recruiting	Fimepinostat / Surgery	OTHER + OP	US, Europe (CH)	https://ClinicalTrials.gov/show/NCT03893487
NCT02208362	Phase 1	Active, not recruiting	IL13Ralpha2-specific Hinge-optimized 4-1BB-co-stimulatory CAR/Truncated CD19-expressing Autologous TN/MEM Cells	IT	US	https://ClinicalTrials.gov/show/NCT02208362
NCT04730349	Phase 1/2	Recruiting	Nivolumab / NKTR-214	IT	US, AU, CA, Europe (FR, DE, IT, NL, ES, GB)	https://ClinicalTrials.gov/show/NCT04730349
NCT02644291	Phase 1	Recruiting	Mebendazole	OTHER	US	https://ClinicalTrials.gov/show/NCT02644291
NCT03605550	Phase 1	Recruiting	PTC596 / Radiotherapy	TT + RT	US	https://ClinicalTrials.gov/show/NCT03605550
NCT02020720	Early Phase 1	Active, not recruiting	Fluorine F 18 Fluorodopa / Biopsy / Therapeutic Surgery / CT / MRI / PET	OP	US	https://ClinicalTrials.gov/show/NCT02020720
NCT03233204	Phase 2	Active, not recruiting	Olaparib	TT	US, PR	https://ClinicalTrials.gov/show/NCT03233204
NCT02457845	Phase 1	Active, not recruiting	G207	IT	US	https://ClinicalTrials.gov/show/NCT02457845

Supplementary Table S2: **Ongoing early phase clinical trials including paediatric patients with recurrent/progressing pHGG (n=56)**
(ClinicalTrials.gov, EudraCT; status: ongoing; 8 March 2022)

ID	Phase	Status	Intervention	Type	Country	Link
NCT03213652	Phase 2	Recruiting	Ensartinib	TT	US	https://ClinicalTrials.gov/show/NCT03213652
NCT03213665	Phase 2	Active, not recruiting	Tazemetostat	TT	US, PR	https://ClinicalTrials.gov/show/NCT03213665
NCT03434262	Phase 1	Recruiting	Gemcitabine / Ribociclib / Sonidegib / Trametinib / Filgrastim	TT + CT	US	https://ClinicalTrials.gov/show/NCT03434262
NCT03213704	Phase 2	Recruiting	Larotrectinib Sulfate	TT	US, PR	https://ClinicalTrials.gov/show/NCT03213704
NCT03526250	Phase 2	Recruiting	Palbociclib	TT	US, PR	https://ClinicalTrials.gov/show/NCT03526250
NCT03220035	Phase 2	Active, not recruiting	Vemurafenib	TT	US, PR	https://ClinicalTrials.gov/show/NCT03220035
NCT03749187	Phase 1	Recruiting	PARP Inhibitor BGB-290 / Temozolomide	TT + CT	US	https://ClinicalTrials.gov/show/NCT03749187
NCT03257631	Phase 2	Active, not recruiting	Pomalidomide	OTHER	US, Europe (FR, IT, ES, GB)	https://ClinicalTrials.gov/show/NCT03257631
NCT03210714	Phase 2	Recruiting	Erdafitinib	TT	US, PR	https://ClinicalTrials.gov/show/NCT03210714
NCT01884740	Phase 1/2	Recruiting	SIACI of Erbitux and Bevacizumab	TT (Intervent.)	US	https://ClinicalTrials.gov/show/NCT01884740
NCT03245151	Phase 1/2	Active, not recruiting	Lenvatinib / Everolimus	TT	US, CA	https://ClinicalTrials.gov/show/NCT03245151
NCT04238819	Phase 1	Recruiting	Abemaciclib / Irinotecan / Temozolomide	TT + CT	US, JP, Europe (BE, FR, DE, IT, ES)	https://ClinicalTrials.gov/show/NCT04238819
NCT04195555	Phase 2	Recruiting	Ivosidenib	TT	US, PR	https://ClinicalTrials.gov/show/NCT04195555
NCT03478462	Phase 1	Recruiting	CLR 131	Targeted RT	US, AU, CA	https://ClinicalTrials.gov/show/NCT03478462
NCT04521946	Phase 1	Recruiting	Etoposide / Fludarabine / Thiotepa / Melphalan / Hematopoietic Cell Transplantation / Lapine T-Lymphocyte Immune Globulin / MMF / Tacrolimus	CT + IT	US	https://ClinicalTrials.gov/show/NCT04521946
NCT04320888	Phase 2	Recruiting	Selpercatinib	TT	US, PR	https://ClinicalTrials.gov/show/NCT04320888
NCT04284774	Phase 2	Recruiting	Tipifarnib	TT	US, PR	https://ClinicalTrials.gov/show/NCT04284774
NCT05135975	Phase 2	Recruiting	Cabozantinib	TT	US	https://ClinicalTrials.gov/show/NCT05135975
EudraCT: 2020-000854-85	Phase 1/2	Ongoing	Bempegaldesleukin / Nivolumab	IT	Europe (FR, DE, IT, ES)	https://www.clinicaltrialsregister.eu/ctr-search/search?query=eudract_number:2020-000854-85
EudraCT: 2018-000096-32	Phase 1/2	Ongoing	Nivolumab and metronomic chemotherapy	IT + CT	Europe (BE, FR)	https://www.clinicaltrialsregister.eu/ctr-search/search?query=eudract_number:2018-000096-32
EudraCT: 2009-016080-11		Ongoing	Gemcitabine / Thiotepa / Etoposide / Melphalan / Irinotecan / Carboplatin / Bevacizumab / Erlotinib / Everolimus	CT + TT	Europe (NL)	https://www.clinicaltrialsregister.eu/ctr-search/search?query=eudract_number:2009-016080-11
EudraCT: 2017-002762-44	Phase 1/2	Ongoing	Pegylated recombinant human Arginase (BCT-100)	OTHER	Europe (IE, NL, GB)	https://www.clinicaltrialsregister.eu/ctr-search/search?query=eudract_number:2017-002762-44
EudraCT: 2014-001929-32	Phase 2	Ongoing	Erlotinib / Everolimus / Dasatinib	TT	Europe (DK, FR, NL, ES, SE, GB)	https://www.clinicaltrialsregister.eu/ctr-search/search?query=eudract_number:2014-001929-32

Supplementary Table S2: **Ongoing early phase clinical trials including paediatric patients with recurrent/progressing pHGG (n=56)** (ClinicalTrials.gov, EudraCT; status: ongoing; 8 March 2022)

ID	Phase	Status	Intervention	Type	Country	Link
EudraCT: 2014-004685-25	Phase 1/2	Active, not recruiting	Cobimetinib	TT	Europe (DK, FR, DE, IE, IT, NL, ES, GB)	https://www.clinicaltrialsregister.eu/ctr-search/search?query=eudract_number:2014-004685-25
EudraCT: 2021-001375-16		Ongoing		TT	Europe (PL)	https://www.clinicaltrialsregister.eu/ctr-search/search?query=eudract_number:2021-001375-16
EudraCT: 2019-000212-28	Phase 1/2	Ongoing	LOXO-292	TT	Europe (DK, FR, DE, IT, ES)	https://www.clinicaltrialsregister.eu/ctr-search/search?query=eudract_number:2019-000212-28
EudraCT: 2014-004438-24	Phase 2	Ongoing	Depatuxizumab mafodotin (ABT-414) / Temozolomide / CCNU	TT + CT	Europe (AT, BE, CZ, FI, FR, DE, HU, IE, IT, NL, PL, ES, GB)	https://www.clinicaltrialsregister.eu/ctr-search/search?query=eudract_number:2014-004438-24
EudraCT: 2016-003498-16	Phase 1/2	Ongoing	LOXO-101	TT	Europe (CZ, DK, FR, DE, IE, IT, NL, PL, ES, SE, GB)	https://www.clinicaltrialsregister.eu/ctr-search/search?query=eudract_number:2016-003498-16

Supplementary Table S2. Ongoing early phase clinical trials including paediatric patients with recurrent/progressing pHGG (n=56).

The online search was conducted on 8 March 2022 at ClinicalTrials.gov (https://clinicaltrials.gov) (n=46) and the EU Clinical Trials Register (https://www.clinicaltrialsregister.eu) (n=10).

Search terms: “HGG” OR “High-grade glioma” AND “relapsed” OR “recurrent”; advanced search: children; (not yet) recruiting; enrolling by invitation; active, not recruiting.

Of the 56 trials, n=44 (79%) are being conducted (mainly) at US sites, and n=17 (30%) involved sites in European countries. Most trials investigate targeted treatment (n=29, 52%) or immunotherapeutic approaches (n=16, 29%) alone or combined with conventional treatment modalities.

Abbreviations: IT, immunotherapy; CT, chemotherapy; TTF, tumour-treating fields; TT, targeted treatment; RT, radiotherapy; OP, surgery.
