# Supplementary material for: How I treat recurrent pediatric high-grade glioma (pHGG): a Europe-wide survey study
Source: J Neurooncol. 2023 Feb 1;161(3):525–38. doi: 10.1007/s11060-023-04241-6 (PMC9992031; doi:10.1007/s11060-023-04241-6)
Supplement: Supplementary file 3 — Supplementary file3 (PDF 177 KB) [file 11060_2023_4241_MOESM3_ESM.pdf]

**Supplementary Table S3. Responses by country of employment (Germany vs. other countries).**

| Treatment | Answers total | Germany (%) | Other Countries (%) | p-value |
|---|---|---|---|---|
| Respondents | n=139 | n=69 | n=70 | Fisher's exact |
| Palliative care | 16.4 % | 19.2 | 13.8 | |
| Oncological treatment | 83.6 % | 80.8 | 86.2 | 0.1507 |
| Curative intent | 44.8 % | 42.7 | 46.6 | |
| Non-curative intent | 55.2 % | 57.3 | 53..4 | 0.5218 |
| Phase I/II trials | 88.7 % | 87.7 | 89.5 | 0.6135 |
| <u>Surgery</u> | 43.4 % | 39.4 | 46.8 | 0.1893 |
| Maximal safe resection | 79.9 % | 77.0 | 81.8 | |
| Biopsy | 20.1 % | 23.0 | 18.2 | 0.5355 |
| Molecular workup | 95.3 % | 95.1 | 95.5 | >0.9999 |
| <u>(Re-) Irradiation</u> | 69.5 % | 69.0 | 69.9 | 0.9062 |
| Local radiotherapy | 81.3 % | 76.4 | 85.3 | |
| Craniospinal radiotherapy | 18.7 % | 23.6 | 14.7 | 0.0943 |
| Dose ≤20 Gy | 32.3 % | 34.9 | 30.2 | |
| Dose >20 Gy | 67.7 % | 65.1 | 69.8 | 0.4849 |
| <u>Chemotherapy</u> | 57.6 % | 58.8 | 56.6 | 0.7394 |
| Lomustine (CCNU) | 17.1 % | 12.2 | 21.4 | 0.1248 |
| Monotherapy | 42.4 % | 27.3 | 10.7 | |
| CCNU + Bevacizumab | 24.2 % | 9.1 | 6.8 | |
| CCNU + Temozolomide | 24.2 % | 45.5 | 2.9 | |
| **Procarbazine + CCNU + VCR** | 7.8 % | **1.1** | **13.6** | **0.0009** |
| Temozolomide | 19.2 % | 21.1 | 17.5 | 0.5842 |
| **Etoposide + Trofo. oral** | 7.8 % | **15.6** | **1.0** | **0.0002** |
| Topo./Eto. +/- Cy oral | 7.3 % | 8.9 | 5.8 | 0.5795 |
| **Modified HIT-SKK** | 5.2 % | **8.9** | **1.9** | **0.0472** |
| **Irinotecan + Bevacizumab** | 4.1 % | **0.0** | **7.8** | **0.0076** |
| Vinblastine | 3.6 % | 5.6 | 1.9 | 0.2544 |
| Oral antiangiogenetic comb. | 3.6 % | 2.2 | 4.9 | 0.4521 |
| Other Chemotherapy[a] | 18.1 % | 16.7 | 11.7 | 0.4060 |
| **CT plus HDAC inhibitors** | 12.4 % | **21.1** | **3.9** | **0.0002** |
| **CT plus Chloroquine** | 2.6 % | **5.6** | **0.0** | **0.0208** |
| <u>Targeted therapy</u> | 89.4 % | 88.7 | 90.0 | 0.7224 |
| Depending on findings/trials | 39.0 % | 42.1 | 36.4 | 0.3388 |
| BRAF/MEK inhibitor | 44.4 % | 40.6 | 47.5 | 0.2415 |
| BRAF inhibitor | 35.9 % | 33.3 | 37.7 | |
| MEK inhibitor | 24.4 % | 18.5 | 28.6 | |
| BRAFi + MEKi | 39.7 % | 44.4 | 36.4 | |
| Anti EGFR | 7.5 % | 4.5 | 10.5 | 0.0794 |
| TKI (Erlotinib) | 45.5 % | 40.0 | 47.1 | |
| Nimotuzumab | 31.8 % | 20.0 | 35.3 | |
| EZH2 inhibitor | 3.7 % | 1.5 | 5.6 | 0.1191 |
| CDK inhibitor | 2.0 % | 1.5 | 2.5 | 0.6935 |
| TRK inhibitor | 2.0 % | 0.8 | 3.1 | 0.2276 |
| DRD2 inhibitor (ONC201) | 2.0 % | 2.3 | 1.9 | >0.9999 |
| mTOR inhibitor | 2.0 % | 0.8 | 3.7 | 0.1333 |
| Other targeted therapy[b] | 3.4 % | 3.8 | 3.1 | 0.7584 |
| **Bevacizumab** | 12.2 % | **3.0** | **19.8** | **<0.0001** |
| <u>Immunotherapy</u> | 43.3 % | 48.7 | 38.8 | 0.0747 |
| **Checkpoint inhibitor** | 30.3 % | **39.7** | **17.4** | **0.0051** |
| Nivolumab | 48.8 % | 48.1 | 50.0 | |
| plus Entinostat | 42.9 % | 29.6 | 12.5 | |
| plus Vaccine | 27.9 % | 37.0 | 25.0 | |
| Comb. (Ipilimumab) | 14.0 % | 0.0 | 37.5 | |
| **Tumor vaccine** | 16.2 % | **26.0** | **5.8** | **0.0051** |
| Dendritic cells | 52.2 % | 52.6 | 50.0 | |
| Other immunotherapy[c] | 4.9 % | 4.1 | 5.8 | 0.7131 |
| Tumor-Treating Fields | 15.9 % | 16.0 | 15.8 | 0.0999 |
| **Intraventricular CT** | 12.9 % | **17.5** | **9.0** | **0.0305** |

**Supplementary Table S3. Responses by country of employment (Germany vs. other countries).**

Chosen answers and free-text proposals by the respondents from Germany vs. other European countries. Percentage is related to the respective number of answers for each question. Bold font indicates a statistically significant difference.

a “Metronomic chemotherapy” not specified, 2.1%; HIT-HGG-Rez-Immunovac study, 2.1%; LGG chemotherapy (carboplatin, vincristine), 2.1%; vincristine or vinorelbine, 2.1%; PEI (cisplatin, etoposide, ifosfamide), 1.6%; carboplatin + etoposide, 1.6%; High-dose chemotherapy + autologous stem cell transfusion, 1.0%; RIST (rapamycin, irinotecan, dasatinib, temozolomide), 1.0%; ICE (ifosfamide, carboplatin, etoposide), 1.0%; carboplatin-based, 0.5%; eribulin, 0.5%; fotemustine, 0.5%; Metro-PD1, 0.5%; adriamycine, 0.5%; vindesine, 0.5%, VAC (vincristine, actinomycin-d, cyclophosphamide), 0.5%;

b Hedgehog inhibitor, 0.7%; PDGFR inhibitor, 0.3%; PIK3CA inhibitor, 0.3%; PARP inhibitor, 0.3%; ALK inhibitor, 0.3%;

c CAR-T cells, 3.5%; Oncolytic virus, 1.4%;

d BRAFV600E mutation and SMARCB1 deletion;

e EGFR overexpression and CDKN2A deletion;

f KIAA1549-BRAF fusion

*Abbreviations:* VCR, vincristine; Trofo, trofosfamide; Topo, topotecan; Eto, etoposide; Cy, cyclophosphamide; comb., combination; CT, chemotherapy; TKI, tyrosine kinase inhibitor.
